# A Type 2 Ryanodine Receptor Variant in the Helical Domain 2 Associated with an Impairment of the Adrenergic Response

**DOI:** 10.3390/jpm11060579

**Published:** 2021-06-20

**Authors:** Malorie Blancard, Zahia Touat-Hamici, Yuriana Aguilar-Sanchez, Liheng Yin, Guy Vaksmann, Nathalie Roux-Buisson, Véronique Fressart, Isabelle Denjoy, Didier Klug, Nathalie Neyroud, Josefina Ramos-Franco, Ana Maria Gomez, Pascale Guicheney

**Affiliations:** 1 Inserm, UMRS 1166, Institute of Cardiometabolism and Nutrition (ICAN), Sorbonne Université, 75013 Paris, France; ztouat@gmail.com (Z.T.-H.); nathalie.neyroud@sorbonne-universite.fr (N.N.); pascale.guicheney@sorbonne-universite.fr (P.G.); 2 Department of Physiology & Biophysics, Rush University Medical Center, Chicago, IL 60612, USA; yuriana.aguilar-torres@bcm.edu (Y.A.-S.); josefina_ramos-franco@rush.edu (J.R.-F.); 3 Inserm, UMRS 1180, Université Paris Saclay, 92290 Châtenay-Malabry, France; liheng.yin@universite-paris-saclay.fr (L.Y.); ana-maria.gomez@inserm.fr (A.M.G.); 4 Service de Cardiologie Pédiatrique, Hôpital Privé de la Louvière, 59042 Lille, France; guy.vaksmann@wanadoo.fr; 5 Grenoble Institut Neurosciences, Université Joseph Fourier, 38400 Grenoble, France; NRouxbuisson@chu-grenoble.fr; 6 Unité de Cardiogénétique et Myogénétique, AP-HP, 75651 Paris, France; vero.fressart@psl.aphp.fr; 7 Département de Cardiologie, Centre de Référence des Maladies Cardiaques Héréditaires, Hôpital Bichat, AP-HP, 75018 Paris, France; isabelle.denjoy@aphp.fr; 8 Hôpital Cardiologique, CHRU de Lille, 59000 Lille, France; didier.klug@chru-lille.fr

**Keywords:** arrhythmia, CPVT, RYR2, calcium, sudden death, adrenergic stimulation

## Abstract

Catecholaminergic polymorphic ventricular tachycardia (CPVT) is triggered by exercise or acute emotion in patients with normal resting electrocardiogram. The major disease-causing gene is *RYR2*, encoding the cardiac ryanodine receptor (RyR2). We report a novel *RYR2* variant, p.Asp3291Val, outside the four CPVT mutation hotspots, in three CPVT families with numerous sudden deaths. This missense variant was first identified in a four-generation family, where eight sudden cardiac deaths occurred before the age of 30 in the context of adrenergic stress. All affected subjects harbored at least one copy of the *RYR2* variant. Three affected sisters were homozygous for the variant. The same variant was found in two additional CPVT families. It is located in the helical domain 2 and changes a negatively charged amino acid widely conserved through evolution. Functional analysis of D3291V channels revealed a normal response to cytosolic Ca^2+^, a markedly reduced luminal Ca^2+^ sensitivity and, more importantly, an absence of normal response to 8-bromo-cAMP and forskolin stimulation in both transfected HEK293 and HL-1 cells. Our data support that the D3291V-RyR2 is a loss-of-function RyR2 variant responsible for an atypical form of CPVT inducing a mild dysfunction in basal conditions but leading potentially to fatal events through its unresponsiveness to adrenergic stimulation.

## 1. Introduction

Catecholaminergic polymorphic ventricular tachycardia (CPVT) is a rare inherited life-threatening disorder characterized by adrenergic induced bi-directional or polymorphic ventricular tachycardia (PVT) in absence of any detectable structural heart abnormalities [1]. It mostly occurs in children and young adults and causes syncope and sudden cardiac death (SCD) [2]. The baseline electrocardiogram (ECG) is usually normal, and diagnosis is therefore based on the occurrence of arrhythmias during exercise-stress testing (EST) or Holter monitoring [3]. In the absence of treatment, CPVT leads to a high mortality rate, especially in probands, and treatment by β-blockers is mandatory [4,5,6]. Flecainide is considered as the first adjuvant treatment to β-blockers when control of arrhythmias is incomplete [7,8].

Missense mutations in *RYR2*, the gene encoding the cardiac ryanodine receptor type 2 (RyR2), are the major cause of the CPVT dominant form with detection of a *RYR2* mutation in 60 to 65% of patients, including a high rate of de novo mutations [9,10,11,12,13]. More recently, several dominant mutations in the calmodulin genes (*CALM1*, *CALM2*, *CALM3*) leading to severe de novo or familial CPVT have been discovered [14,15,16,17,18]. Recessive mutations account for a small fraction of CPVT patients and have been mainly associated with proteins regulating the RyR2 complex activity such as calsequestrin2 (*CASQ2*), triadin (*TRDN)* and trans-2,3-enoyl-CoA reductase-like (*TECRL*) [19,20,21]. 

RyR2 is a large homotetrameric channel located in the sarcoplasmic reticulum (SR) membrane. It handles the Ca^2+^ release from the sarcoplasmic reticulum (SR) to the cytosol, an essential process in the cardiac excitation-contraction coupling (ECC). Another mechanism occuring in cardiomyocytes termed ‘store-overload induced Ca^2+^ release’ (SOICR) has been described as the spontaneous release of Ca^2+^ through RyR2 that occurs when the SR Ca^2+^ content reaches a critical level [22,23]. The SOICR results in sparks in cardiomyocytes and is displayed in HEK293 cells expressing RyR2, as spontaneous oscillations, in a manner virtually identical to that observed in cardiac cells [24]. 

The extent of RyR2 variants responsible for cardiac arrhythmias and cardiomyopathies as well as the various underlying pathogenic mechanisms remains unknown [10,25,26,27]. The typical CPVT RyR2 mutations render RyR2 channels more prone to spontaneous Ca^2+^ release resulting in Ca^2+^ waves that trigger membrane depolarizations, premature ventricular beats (PVB), and polymorphic ventricular tachycardia (PVT) during physical activity or emotional stress [18,25]. These gain of function (GOF) mutations are mostly located in four specific domains [28,29]. In addition, loss of function (LOF) RyR2 mutations have also been identified and recently linked to a distinct entity of cardiac arrhythmia termed ‘RyR2 Ca^2+^ release deficiency syndrome’ (CRDS) [27,30,31,32,33,34]). The CRDS patients show a normal exercise stress test (EST) and display syncope and sudden death due to ventricular fibrillation [31]). 

In this study, we report a novel *RYR2* variant, located outside the mutation hotspots, found in three families with atypical CPVT. The largest family was a four-generation one with homozygous and heterozygous carriers of the variant, where eight family members died suddenly before the age of 30, in the context of acute emotion or exercise. Here, we assessed the Ca^2+^ release activity of the RyR2 variant, as well as its response to adrenergic stimulation at the cellular and single channel level. 

## 2. Materials and Methods

### 2.1. Patients

CPVT was suspected in three families due to a history of sudden death in young family members during physical exercise or emotional stress. Clinical evaluation of the patients included 12-lead ECG, 24 h Holter ECG, EST, isoprenaline infusion and echocardiography. Relatives were considered affected when an EST or isoprenaline test showed bi-directional premature ventricular beats (PVBs), bigeminy, bi-directional or PVT [3]. 

Blood samples were collected for genetic analyses after obtaining signed consent for a study approved by the local ethics committee of the Saint-Louis Hospital (Paris, France). The study was conducted according to the principles of the Helsinki Declaration.

### 2.2. Genetic Analysis 

Genomic DNA was isolated from whole blood using a standard protocol. All coding exons of *RYR2* (NM_001035), *CASQ2* (NM_001232), and *TRDN* (NM_006073) were screened by direct Sanger sequencing and linkage analysis was performed in family 1 with intragenic or closely linked microsatellites to the *RYR2* locus (D1S2800, D1S2680, D1S204). Several genes (*CASQ2*, *CALM1*, *RYR2*, *TRDN*, *SCN5A*, *KCNQ1*, *DSC2*, *DSG2*, *DSP*, *KCNE1*, *KCNE2*, *KCNH2*, *KCNJ2*, *LMNA*, *NKX2-5*, *PKP2*) implicated in arrhythmias were screened for the proband of family 2 by Multiplex Amplification of coding regions and then analyzed by high-throughput sequencing. The *RYR2* variant was genotyped by Sanger sequencing in all family members.

### 2.3. RYR2 Cloning and Mutagenesis

The plasmid containing the human RyR2 sequence (X98330) tagged with enhanced green fluorescent protein (eGFP) was kindly provided by Dr. Spyros Zissimopoulos (UK) [35,36] and corrected as described previously [37]. To generate the D3291V variant, a fragment of hRyR2, digested by KpnI and FseI, was sub-cloned into pCR^®^-Blunt, and the aspartic acid was replaced by a valine by site-directed mutagenesis. The mutated KpnI/FseI fragment of the pCR^®^-Blunt plasmid was then amplified by overlapping PCR to be re-inserted into the full-length RYR2 plasmid using a recombination kit (NEBuilder HiFi DNA Assembly Cloning kit, New England Biolabs, Ipswich, MA, USA). A GFP-free hRyR2 plasmid was prepared for single channel experiments using a recombination kit (NEBuilder HiFi DNA Assembly Cloning kit, New England Biolabs, Evry, France). The GFP sequence was removed from the full-length RyR2 plasmid by AfeI/FspAI digestion. This plasmid was then sealed with a GFP-free RyR2 PCR fragment with overlapping ends to the linearized AfeI/FspAI digested RyR2 plasmid.

### 2.4. Cell Culture and Transfection

Human embryonic kidney 293 (HEK293) cells were cultured in Dulbecco’s Modified Eagle Medium (DMEM) (Life Technologies, Villebon-sur-Yvette, France) supplemented with 10% FBS and 1% penicillin/streptomycin. Lipofectamine 3000 (Life Technologies, Saint-Aubin, France) was used to transfect 60–70% confluent HEK293 cells with either eGFP-WT-hRyR2 or eGFP-D3291V-hRyR2 plasmids. 

HL-1 cardiac cells (Merck, Lyon, France) were grown in culture flasks coated with 0.02% gelatin and 10 μg/mL fibronectin, and were maintained in Claycomb media (Sigma Aldrich, St. Quentin Fallavier, France) supplemented with 10% FBS, 1% penicillin/streptomycin, 2 mM L-glutamine, and 0.1 mM norepinephrine. For transfection, HL-1 cells were cultured into 35 mm µ-dish with glass bottom (Ibidi, Clinisciences, France), coated with gelatin and fibronectin. Cells were grown up to ~30% confluency and then transfected using X-tremGENE HP DNA Transfection Reagent (Roche, Meylan, France) according to manufacturer’s instructions. Briefly, 1 μg of eGFP-WT-hRyR2 or eGFP-D3291V-hRyR2 plasmids was mixed with 100 μL of FBS and antibiotic free OPTI-MEM medium, followed by addition of 2 μL X-tremGENE and incubated at room temperature for 20 min. Culture medium in 35 mm µ-dishes was replaced with fresh Claycomb media, and the transfection solution was then applied directly to the cells and incubated at 37 °C for 48 h. Using this method, the transfection efficiency of HL-1 cells was ~20–30%. 

### 2.5. Protein Extraction and Western Blotting

HEK293 cells were seeded in 25 cm^2^ dishes and then transfected with 2 µg of eGFP-WT-hRyR2 or eGFP-D3291V-hRyR2 plasmids. Forty-eight hours after transfection, cells were treated with either 0 μM or 250 μM of 8-bromo-cAMP (cAMP) (Sigma-Aldrich, France) for 20 min and were then lysed with a buffer containing 50 mM Tris-HCl (pH 7.5), 150 mM NaCl, 1% Triton X-100, 0.5% sodium deoxycholate, 0.1% SDS and 5 mM EDTA. Cell lysates were incubated for one hour at 4 °C under gentle stirring and then centrifuged at 16,000 rpm for 10 min at 4 °C. Protein concentrations were determined using a Pierce^TM^ BCA Protein Assay kit (ThermoScientific, Illkirch-Graffenstaden, France). Total proteins (20 µg) were separated by 3–8% Tris Acetate polyacrylamide gel electrophoresis and then transferred into a nitrocellulose membrane for 90 min at 35 V. Membranes were saturated with 5% milk-0.1% Tween-Phosphate Buffered Saline (PBS) for 45 min and then incubated overnight with antibodies against RyR2 (1/1000) (MA3-916, ThermoScientific, France) and α-tubulin (1/2000) (ab4074, Abcam, Cambridge, UK) Membranes were washed four times in 0.1% Tween-PBS and then incubated with IR-fluorescent secondary antibodies (1/10,000) (ThermoScientific, France). Membranes were washed again twice in 0.1% Tween-PBS and twice in PBS before IR-fluorescence imaging. RyR2 and α-tubulin expression was quantified with the ImageJ software (NIH, Bethesda, MD, USA).

For S2808 phosphorylation assessment, membranes were then stripped for 10 min using Restore^TM^ PLUS Western Blot Stripping Buffer (Thermo Scientific, France) and then washed with PBS. Membranes were saturated again with 5% milk-0.1% Tween-Phosphate Buffered Saline (PBS) for 45 min and then incubated overnight with antibodies against pSer2808-RyR2 (1/1000) (Badrilla, France) and α-tubulin (1/2000). Membranes were washed four times in 0.1% Tween-PBS and then incubated with HRP secondary antibodies (1/5000) (Cell Signaling, Paris, France). Membranes were washed again four times in 0.1% Tween-PBS and then revealed using a chemoluminescent kit, SuperSignal^TM^ West Femto Maximum Sensitivity Substrate (ThermoScientific, France).

### 2.6. Calcium Imaging

HEK293 and HL-1 cells were seeded in 35 mm µ-dish with glass bottom (Ibidi, Clinisciences, France). After 48 h, transfected HEK293 and HL-1 cells (280 ng plasmid/dish) were loaded with the fluorescent Ca^2+^ dye Fura2-AM (5 µM) for 45 min at 37 °C, 5% CO_2_ in Krebs–Ringer–Hepes (KRH) buffer composed of (in mM): 125 NaCl, 25 HEPES, 5 KCl, 1.2 MgCl_2_, 6 glucose, pH 7.4. Cells were then perfused with KRH buffer containing different Ca^2+^ concentrations (0.1, 0.5 or 1 mM) at room temperature. At the end of each measurement, 10 mM caffeine was added in order to select only the responsive cells. Single-cell intracellular Ca^2+^ measurements were performed in Fura-2-loaded cells using a Nikon Eclipse Ti-U inverted fluorescent microscope (Nikon France, Champigny-sur-Marne, France) equipped with a dual excitation filter wheel (340 nm/380 nm), a 515 nm emission filter and a Hamamatsu ORCA-D2 CCD camera (Hamamatsu Photonics France, Massy, France). Ca^2+^ traces were obtained by sequential acquisition of one image every 2 s for 3 min using a 20X S-Fluor 0.75 NA (WD = 1mm) objective. Image acquisition and analysis were performed with the HCimage software (Hamamatsu, Sewickley, PA, USA). Fluorescence intensities in individual cells were determined using region of interest (ROI) analysis with ImageJ software (National Institutes of Health). Ca^2+^ signals were measured in the caffeine-responsive cells using F340/380 ratios after subtraction of Fura-2 background fluorescence. For experiments with cAMP treatment, cells were pretreated with 250 µM of 8-bromo-cyclic AMP (Sigma-Aldrich, France) for 20 min. at room temperature before Ca^2+^-KRH perfusion. 

### 2.7. Single Channel Analysis 

Single-channel studies were carried out with crude microsomes obtained from HEK293 cells transfected with WT-hRyR2 or D3291V-hRyR2 plasmids. Lipid planar bilayers were made of a mixture of phosphatidylethanolamine, phosphatidylserine, and phosphatidylcholine in a 5:4:1 ratio (50 mg/mL in decane) (Avanti Polar Lipids Inc., Alabaster, AL, USA). Microsomes were incorporated into the lipid bilayers formed across a 100 μm hole in a thin teflon partition separating two aqueous compartments. To examine the cytosolic Ca^2+^-dependency of single channel open probability (Po), the cytosolic compartment (cis chamber) was virtually grounded and filled with a 250 mM HEPES and 120 mM Tris solution at pH 7.4, and then, microsomes were added to the cis chamber. The luminal compartment (trans chamber) was filled with 250 mM HEPES and 50 mM Ca^2+^ at pH 7.4, and the voltage was applied in this compartment. To examine the luminal Ca^2+^-dependency, the solution in cis and trans contained 250 mM CsCH_3_SO_3_, 0.755 mM CaCl_2_, 1 mM BAPTA, 20 mM HEPES, pH 7.4. Immediately upon observing single-channel activity, we replaced the solutions to establish the specific free Ca^2+^ concentrations. Ca^2+^-buffer solution (BAPTA and 5-5′-dibromo-BAPTA, Thermo Fisher Scientific, Waltham, MA, USA) compositions were calculated using the MaxChelator software. Membrane voltage was controlled using an Axopatch 200B (Molecular Devices LLC, San Jose, CA, USA). The current signal was digitized at 20 kHz through a Digidata 1322A interface (Molecular Devices LLC, USA) and subsequently filtered at 2 kHz, unless specified otherwise. Data acquisition and analysis were carried out using *pClamp* software (Molecular Devices LLC, USA). 

### 2.8. Statistical Analysis

Cellular data and single channel results are expressed as mean ± SEM unless otherwise stated. Results for fitting histograms and the Hill equation are given as the mean ± SD. Statistical tests (unpaired Student’s *t*-test, ANOVA) were performed with OriginPro Software (OriginLab, Northampton, MA, USA). A *p*-value of 0.05 was considered significant.

## 3. Results

### 3.1. Clinical Phenotype and Genetic Analysis

#### 3.1.1. Family 1

**Figure 1:**
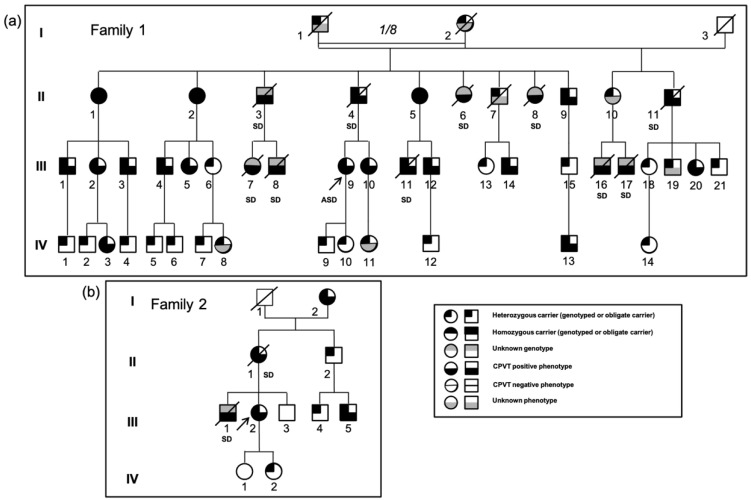
Pedigrees of two families. (**a**) Pedigree of Family 1; (**b**) Pedigree of Family 2. Squares and circles indicate males and females, respectively, and slashes indicate deceased individuals. For Family 1, only deceased and living D3291V carriers were indicated; thirty healthy genotype negative carriers were excluded. Genotypes and phenotypes are indicated with black and grey boxes. SD: Sudden death, ASD: Aborted Sudden Death.

**Figure 2:**
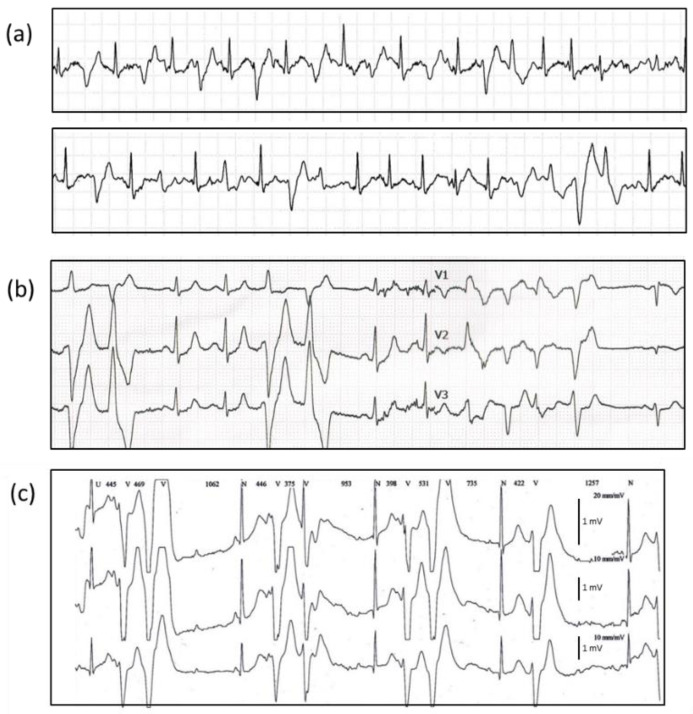
Abnormal ECGs during ESTs in D3291V heterozygous carriers. (**a**) EST performed in an asymptomatic untreated subject (III.14, family 1) after genetic diagnosis at the age of 17 showing bigeminy, bi-directional doublets, and ventricular tachycardia at 125 W; (**b**) Polymorphic ventricular tachycardia during EST in a 44-year-old subject treated with 80 mg of nadolol (III.2, family 1). Her daughter (IV.3) was also treated with 80mg of nadolol and developed PVT; (**c**) Holter recording of an untreated child (IV.13, family 1) at the age of 2 with AVB and bi-directional ventricular doublets at 110 bpm.

Family 1 is a four-generation consanguineous family originating from Northern France in which eight sudden deaths occurred during physical or emotional stress before the age of 30 (Figure 1a, Table S1). The grandparents were first cousins. The proband III.9 had her first syncope at the age of 10 and experienced an aborted sudden cardiac death at the age of 14 in the context of emotion and moderate hypokalemia (3.2 mmol/L). Relatives underwent clinical evaluations including a 12-lead ECG, an echocardiography, a 24 h Holter ECG and ESTs or isoprenaline tests. All ECGs were normal at rest, and echocardiograms showed no structural abnormalities. In relatives harboring the variant, before β-blocker treatment, no sinus bradycardia was noted on resting ECG nor attenuated heart rate response or limitation to exercise during EST. Although the majority of family members were taking the β-blocker nadolol, bidirectional ventricular tachycardia or PVT was detected during clinical evaluation mostly in generation II and III members (Figure 2a,b). T wave alternans were not observed but were not assessed with a dedicated software on ECG or Holter monitoring. Thus, subtle variations in T wave amplitude could have been missed. Heart rate variability was not examined. However, it should be noted that there were relatively few living symptomatic subjects compared to the high number of sudden deaths.

When the proband was initially diagnosed with CPVT following her cardiac arrest, exons of RyR2 encompassing hot spot mutations and all coding exons of *CASQ2* and *TRDN* were first screened, but no variant was found. Linkage analysis with microsatellite markers was then performed, which showed cosegregation of the *RYR2* locus with the affected phenotype (not shown). Hence, all *RYR2* exons were screened, and a new variant was identified on exon 68, c.9872A>T, leading to a change of an aspartate to a valine at position 3291. This variant, p.Asp3291Val (D3291V), was absent in all databases (Genome Aggregation Database: gnomAD 2.1.1).

In accordance with the microsatellite haplotypes, the variant was found at the homozygous state in three sisters of the second generation (II.1, II.2, II.5) and at the heterozygous state in all of the other positive-phenotype members. Surprisingly, one of the three homozygous sisters (II.5) never had syncope, but her EST test was stopped because of numerous bidirectional PVBs.

Her sister II.1 had exercise-related syncope on several occasions and an isoprenaline test showed bidirectional PVBs and a short run of VT. The last homozygous sister II.2 also had exercise-related syncope at 18 and her EST revealed PVT. Two siblings died suddenly during physical activity at age 24 (II.3) and 17 (II.6) and one sibling died during acute emotional stress at 16 (II.8). A brother (II.4) who was a heterozygous carrier and had coronary disease died while sleeping at the age of 54. The eight children of the three homozygous sisters were all obligate heterozygous carriers, and one of them died while swimming at the age of 22 (III.11). The grandmother (I.2) also had three children from a second marriage. One of them (II.11) died at age 54, and one affected daughter (II.10) lost her two sons (II.16, II.17) when they were 20 and 15 years old while swimming and biking, respectively.

Most of the negative-phenotype members of the second and third generations did not carry the variant, except for six members, III.6, III.13, III.15, III.18, III.20, and III.21, who were heterozygous carriers with a negative EST at the age of 32, 21, 25, 49, 46, and 39, respectively. Two girls (III.5, III.10) had a negative exercise stress-test at first examinations at the age of 11 and 16 but developed bigeminy during EST later at the age of 21 and 27. In the fourth generation, only 2 of the 14 young carriers had a positive phenotype: IV.3 who had bigeminy during an EST even under the administration of nadolol (80 mg) and IV.13 who presented with an atrioventricular block (AVB) and bradycardia at birth and salvos of non-sustained ventricular tachycardia during a Holter recording at the age of 2 (Figure 2c).

#### 3.1.2. Family 2

The proband (III.2) had syncope at age 20 and 25. While on a β-blocker, her EST performed revealed many PVBs and ventricular bigeminy originating from the right ventricular outflow track (RVOT), which disappeared during recovery, but no typical bidirectional PVT was observed. MRI and echocardiography were normal, and the implanted heart monitor did not record any abnormality. Her mother (II.1) died suddenly at age 45 in the context of emotion, attending the funeral of a friend, and her brother (III.1) died at 8 years old while swimming. The proband’s DNA was screened using a genetic testing panel for cardiac diseases and the only variant found as potentially pathogenic was D3291V in *RYR2* at the heterozygous state. The maternal grandmother (I.2) was positive for this variant and remained asymptomatic for her whole life. Nevertheless, a 24 h Holter recording performed at the age of 80 revealed many PVBs and bigeminy. Her echocardiogram was normal. The mother (II.1) who died at age 45 was thus an obligate carrier of the variant.

The Family 1 and Family 2 originate from the same region in Northern France, and they probably share a common ancestor as they harbor the same mutated haplotype (not shown).

#### 3.1.3. Family 3

The same variant was recently identified in a girl from another family from the same geographical area. She died suddenly while swimming at the age of 10 without prior symptoms. No family inquiry has occurred yet.

### 3.2. Position and Conservation of the RyR2 D3291V Variant

**Figure 3:**
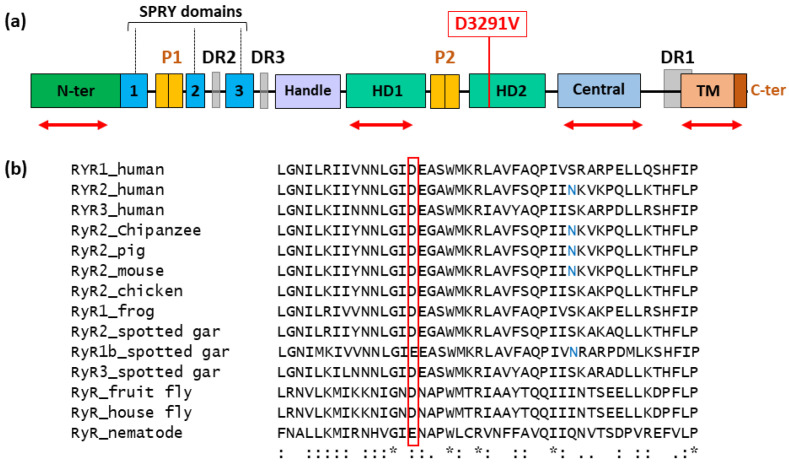
Location of the D3291V-RyR2 variant. (**a**) The D3291V variant is located in the HD2 outside the 4 mutation hotspots (red double arrows) where CPVT mutations are mostly found [39,40]. (**b**) Sequence alignment of RyR orthologues and paralogues. The Uniprot accession numbers are the following: *Homo sapiens* (RyR1 P21817, RyR2 Q92736, RyR3 Q15413), *Pan troglodytes (RyR2* *A0A2I3RIJ4)*, *Sus scrofa* (RyR2 F1RHM3), *Mus musculus* (RyR2 E9Q401), *Gallus gallus* (RyR2 AOA1D5PAZ1), *Xenopus tropicalis* (RyR1 F7E4CO), *Lepisosteus oculatus* (RyR2 W5NIQ4, RyR1 W5NAB3, RyR3 W5N4D0), *Drosophila melanogaster* (RyR Q24498), *Musca domestica* (RyR A0A1I8MDA8), *Caenorhabditis elegans* (I2HAA6_CAEEL, unc-68). Sequence alignment was done at http://www.ebi.ac.uk/Tools/msa/clustalo/ (accessed on 1 January 2018). The Aspartate 3291 is boxed in red. The variant N3308S (in blue) was identified in a CPVT patient [41].

The negatively charged aspartate at position 3291 is located in the helical domain 2 (HD2) of RyR2, where no pathogenic mutation has been reported (Figure 3a). This residue shows a high conservation level through evolution, as well as in RyR1 and RyR3. In vertebrates, the whole area is well conserved with two negatively charged residues next to one another (aspartate-glutamate: D-E). D3291 is also conserved in a number of invertebrates, such as flies. It is replaced by a negatively charged glutamate in *Caenorhabditis elegans* (Figure 3b).

In contrast to RyR1, the C-terminus of the RyR2 HD2 could not be assessed by structural crystallography since number of armadillo repeats in this domain were not visible [38]. This domain is implicated in the conformational changes characterized by the outward tilt of the four S6 segments inducing the dilation of the central pore [38].

### 3.3. Blunted Adrenergic Response of D3291V-RyR2 Channels in HEK293 Cells

**Figure 4:**
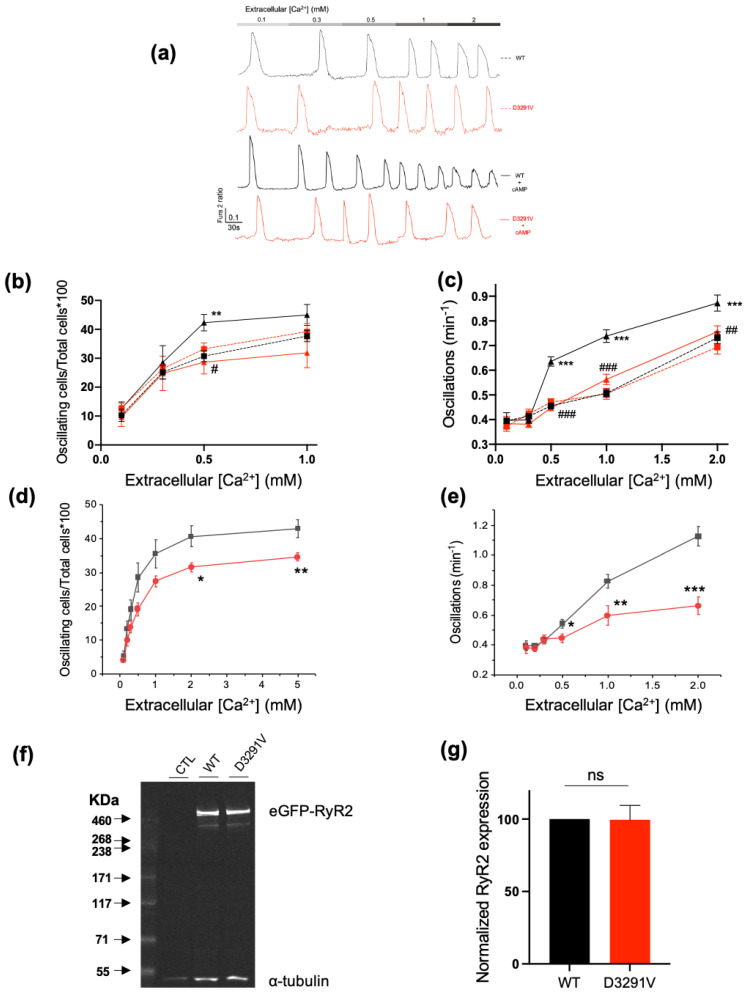
Functional study of D3291V-RyR2 channel in HEK293 cells. (**a**) Representative traces of Ca^2+^ release under basal conditions and after cAMP treatment. (**b**) Percentages of oscillating WT-RyR2 (n = 29 dishes) and D3291V-RyR2 cells (n = 23 dishes) at different Ca^2+^ concentrations (0.1–1 mM) under basal conditions or challenged by 250 µM 8-bromo-cyclic AMP (cAMP). WT: black squares and dotted lines; WT + cAMP: black triangles and solid lines; D3291V: red squares and dotted lines; D3291V + cAMP: red triangles and solid lines. (**c**) Number of oscillations per min of WT-RyR2 (n = 300 cells) or D3291V-RyR2 cells (n = 265 cells) at different Ca^2+^ concentrations (0.1–2 mM) under basal conditions or challenged by 250 µM cAMP. (**d**) Percentages of oscillating WT-RyR2 (n = 29 dishes) (black squares) and D3291V-RyR2 (n = 23 dishes) (red circles) cells at different concentrations of Ca^2+^ (0.1–1 mM) challenged by 5 µM forskolin. (**e**) Number of oscillations per minute of WT-RyR2 (n = 300 cells) (black squares) or D3291V-RyR2 cells (n = 265 cells) (red circles) at different Ca^2+^ concentrations (0.1–2 mM) under basal condition or challenged by 5µM forskolin. (**f**) Representative Western blot of total proteins extracted from HEK293 cells transfected with RYR2-WT or RYR2-D3291V plasmids (n = 12). (**g**) Western blot quantification of RyR2 expression normalized to α-tubulin expression. Statistical significance denoted as WT compared to WT+ cAMP: * *p* < 0.05, ** *p* < 0.01, *** *p* < 0.001. WT + cAMP compared to D3291V + cAMP: ^#^ *p* < 0.05, ^##^ *p* < 0.01, ^###^ *p* < 0.001.

To study the effects of D3291V-RyR2 on channel function, HEK293 cells were transfected with eGFP-WT-hRyR2 or eGFP-D3291V-hRyR2 plasmids. Forty-eight hours after transfection, WT and D3291V-RyR2 expressing cells were loaded with Fura2-AM and then challenged with increasing Ca^2+^ concentrations (0.1 to 1 mM) in order to assess the SOICR activity. The spontaneous Ca^2+^ release was recorded using fluorescence microscopy (Figure 4a). In basal conditions, the percentage of oscillating cells and the number of oscillations per min occurred to be similar between cells expressing WT and D3291V-RyR2 suggesting that the mutant channel has a normal sensitivity to cytosolic Ca^2+^ (at 0.5 mM Ca^2+^, WT = 30.71% ± 1.89 vs. D3291V=33.24% ± 2.07, Figure 4b; WT = 0.45 ± 0.012 vs. D3291V = 0.47 ± 0.015, Figure 4c). The protein expression level of WT-RyR2 and D3291V-RyR2 channels were also similar as shown by Western blots (WT: 100% vs. D3291V: 99.53 ± 9.93%, n = 12, Figure 4f,g).

Because the cardiac arrests were triggered by adrenergic discharges, WT-RyR2 and D3291V-RyR2 transfected cells were challenged by 250 µM 8-Bromo-cyclic AMP (cAMP) or 5 µM forskolin to mimic this condition. As expected, at 0.5 mM Ca^2+^, cAMP treatment induced a higher percent of oscillating WT-RyR2 cells compared to untreated WT-RyR2 cells (WT = 30.71% ± 1.89 vs. WT + cAMP = 42.30% ± 2.84, *p* = 0.008, Figure 4b). This treatment also induced a significant increase in the number of oscillations per minute for WT-RyR2 cells compared to untreated ones at 0.5, 1 and 2 mM Ca^2+^ (WT = 0.45 ± 0.011 vs. WT + cAMP = 0.63 ± 0.019, *p* < 0.001; 0.50 ± 0.010 vs. 0.73 ± 0.025, *p* < 0.001; 0.73 ± 0.02 vs. 0.87 ± 0.03, *p* < 0.001, respectively, Figure 4c).

In contrast, cAMP treatment on D3291V-RyR2 cells did not affect the percentage of oscillating cells nor the number of oscillations per min at any Ca^2+^ concentration. Of note, cAMP treatment revealed significant differences between WT-RyR2 and D3291V-RyR2 cells both for the fractions of oscillating cells at 0.5 mM Ca^2+^ (WT + cAMP = 42.30% ± 2.84 vs. D3291V + cAMP = 28.65% ± 4.08, p = 0.017) and the number of oscillations per minute at 0.5, 1, and 2 Ca^2+^ (for instance at 2 mM Ca^2+^: WT + cAMP = 0.87 ± 0.03 vs. D3291V + cAMP: 0.75 ± 0.02, *p* < 0.001, Figure 4b,c). Similar results were obtained with 5 μM forskolin (Figure 4d,e). Our results suggested that the variant D3291V abolishes the channel response to adrenergic stimulation.

### 3.4. Decreased Phosphorylation Level of D3291V Channels at S2808

**Figure 5:**
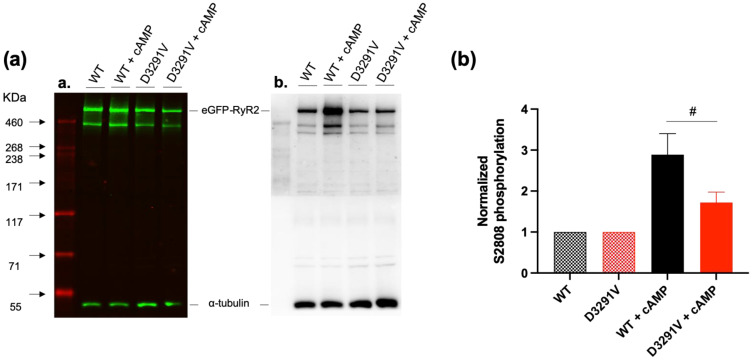
S2808 phosphorylation of both WT and D3291V in presence of cAMP: (**a**) Western blot of total proteins extracted from HEK293 cells transfected with eGFP-WT-hRyR2 or eGFP-D3291V-hRyR2 plasmids and treated or not with 250 μM 8-Bromo-cyclic AMP (cAMP) (n = 6) revealed with anti-RyR2 (A.a.) or anti-pSer2808 (A.b.). (**b**) Quantification of Ser2808 phosphorylation normalized to RyR2 total expression. ^#^ *p* < 0.05, WT + cAMP compared to D3291V + cAMP.

To assess whether the degree of phosphorylation could be responsible for the blunted response of the D3291V-RyR2 to cAMP stimulation, we studied S2808 phosphorylation in both WT and D3291V-RyR2 cells by Western blotting. cAMP induced an increased phosphorylation at S2808 in both WT and D3291V expressing cells but at a lower level in D3291V-RyR2 compared to the WT channels (2.24 ± 0.3 fold in WT vs. 1.46 ± 0.13 fold in D3291V, *p* = 0.042, n = 6, Figure 5). These results suggest that the D3291V variant interferes with phosphorylation of S2808.

### 3.5. Dominant Effect of D3291V Variant in HL-1 Cells

To ascertain the impact of the D3291V variant on RyR2 channel function in the context of cardiac cells, we transfected HL-1 cells, a murine atrial cell line expressing endogenous RyR2, ion channels and all proteins involved in the cAMP pathway, with eGFP-WT-hRyR2 or eGFP-D3291V-hRyR2 plasmids [42].

**Figure 6:**
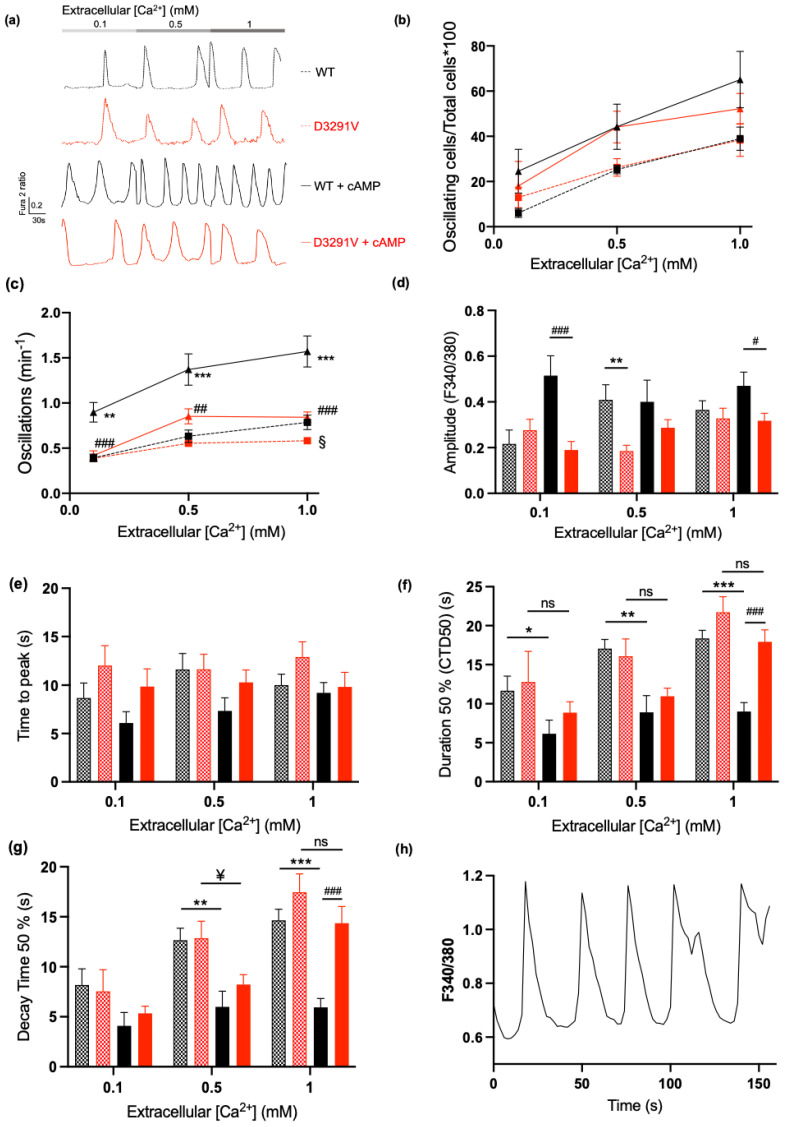
Dominant effect of D3291V in HL-1 cardiomyocytes: (**a**) Representative traces of Ca^2+^ release under basal conditions and after cAMP treatment. (**b**) Percentages of oscillating WT-RyR2 cells (n = 4 dishes) and D3291V-RyR2 cells (n = 5 dishes) at different Ca^2+^ concentrations (0.1, 0.5, 1 mM) under basal conditions or challenged by 250 µM cAMP. WT: black squares and dotted lines; WT + cAMP: black triangles and solid lines; D3291V: red squares and dotted lines; D3291V + cAMP: red triangles and solid lines. (**c**) Number of oscillations per minute of WT-RyR2 (n = 90 cells) or D3291V-RyR2 cells (n = 209 cells) at different Ca^2+^ concentrations (0.1, 0.5, 1 mM) under basal conditions or challenged by 250 µM cAMP. (**d**) Amplitude of the Ca^2+^ released at each spontaneous oscillation at different Ca^2+^ concentrations (0.1–1 mM) under basal conditions or challenged by 250 µM cAMP. (**e**) Time to peak at different Ca^2+^ concentrations (0.1–1 mM) under basal conditions or challenged by 250 µM cAMP. (**f**) Calcium transient duration at 50% (CTD50) at different Ca^2+^ concentrations (0.1–1 mM) under basal conditions or challenged by 250 µM cAMP. (**g**) Decay time 50% at different Ca^2+^ concentrations (0.1–1 mM) under basal conditions or challenged by 250 µM cAMP. (**h**) Representative trace of Ca^2+^ release of D3291V-RyR2 cells after cAMP treatment at 0.5 mM of extracellular Ca^2+^. WT compared to D3291V: ^§^ *p* < 0.05. WT compared to WT + cAMP: * *p* < 0.05, ** *p* < 0.01, *** *p* < 0.001. D3291V compared to D3291V + cAMP: ^¥^ *p* < 0.05. WT + cAMP compared to D3291V + cAMP: ^#^ *p* < 0.05, ^##^ *p* < 0.01, ^###^ *p* < 0.001.

Forty-eight hours post-transfection, WT or D3291V-RyR2 cells were loaded with Fura2-AM and the spontaneous Ca^2+^ release of GFP-positive cells was recorded at increasing Ca^2+^ concentrations (0.1, 0.5, 1 mM) (Figure 6a). In basal conditions, the percentages of oscillating WT and D3291V-RyR2 cells were similar (Figure 6b). Nevertheless, D3291V-RyR2 cells showed a significantly reduced number of oscillations compared to WT-RyR2 cells at 1 mM Ca^2+^ (WT = 0.78 ± 0.08 vs. D3291V = 0.58 ± 0.03, *p* = 0.0249, Figure 6c). The amplitude of the Ca^2+^ release in D3291V-RyR2 cells was significantly decreased only at 0.5 mM Ca^2+^ (WT = 0.4090 ± 0.07 vs. D3291V = 0.1850 ± 0.025, *p* = 0.0048, Figure 6d). The Ca^2+^ transient kinetics—time to peak, duration at 50% (CTD50) and decay time at 50%—were similar for WT and D3291V-RyR2 cells (Figure 6e–g).

Under cAMP stimulation, as expected, we observed a higher percent of oscillating WT-RyR2 HL-1 cells with a higher number of oscillations per min at all Ca^2+^ concentrations (Figure 6b,c). In the same conditions, D3291V-RyR2 HL-1 cells revealed a low increase in the number of oscillations per minute at 0.5 and 1 mM Ca^2+^ compared to untreated D3291V-RyR2 HL-1 cells (Figure 6c). Thus, WT-RyR2 and D3291V-RyR2 treated cells showed significant differences in the number of oscillations per minute at all concentrations (WT + cAMP = 0.89 ± 0.11 vs. D3291V + cAMP = 0.42 ± 0.05, *p* = 0.0001; 1.37 ± 0.17 vs. 0.85 ± 0.08, *p* < 0.0027; 1.57 ± 0.17 vs. 0.84 ± 0.06 *p* < 0.0001, at 0.1, 0.5, and 1 mM Ca^2+^ respectively, Figure 6c). Under these conditions, D3291V-RyR2 HL-1 cells showed a 63% and 32% decrease in the amplitudes of Ca^2+^ release at 0.1 mM Ca^2+^ and 1 mM Ca^2+^, respectively compared to WT-RyR2 cells (WT + cAMP = 0.515 ± 0.087 vs. D3291V + cAMP = 0.189 ± 0.037, *p* = 0.0006; 0.4701 ± 0.06 vs. 0.318 ± 0.032, *p* = 0.037; at 0.1 and 1 mM Ca^2+^, respectively, Figure 6d). The time to peak was similar between WT and D3291V-RyR2 cells after cAMP treatment (Figure 6e). Under cAMP stimulation, as expected, WT-RyR2 cells showed a 47.2%, 47.8% and 50.9% reduction of the CTD50 at 0.1, 0.5, and 1 mM Ca^2+^, respectively (Figure 6f, *p* = 0.046, *p* = 0.004 and *p* < 0.000001 at 0.1, 0.5, and 1 mM Ca^2+^, respectively). Nevertheless, D3291V-RyR2 HL-1 cells did not show a significative reduction of the CTD50 at all concentrations (Figure 6f). This lack of cAMP response from D3291V-RyR2 cells after cAMP treatment induces a significant difference between WT and D3291V-RyR2 cells at 1 mM Ca^2+^ (WT + cAMP = 9.008 ± 1.139 vs. D3291V + cAMP = 17.93 ± 1.54, *p* = 0.00013). As expected, after cAMP treatment, the decay time 50% measurements of WT-RyR2 cells revealed a 52.8% and 59.4% reduction at 0.5 and 1 mM Ca^2+^, respectively (Figure 6f, *p* = 0.003 and *p* = 0.000001 at 0.5 and 1 mM Ca^2+^). D3291V-RyR2 HL-1 cells showed a significant reduction of the decay time compared to untreated D3291V-RyR2 HL-1 cells only after cAMP treatment at 0.5 mM Ca^2+^ (36%) (Figure 6f). It is interesting to note that even if we have a significant reduction of the decay time for D3291V-RyR2 HL-1 cells under cAMP treatment, this reduction is less that the one observed in WT-RyR2 HL-1 cells (36% reduction in D3291V-RyR2 HL-1 cells vs. 52.8% in WT-RyR2 HL-1 cells). Additionally, under cAMP stimulation, early after depolarizations (EADs) were detected in 6% of the D3291V-RyR2 HL-1 cells at 0.5 mM (Figure 6h) while no EADs were detected in WT-RyR2 HL-1 cells at any Ca^2+^ concentration. No alternans were observed in D3291V-RyR2 HL-1 cells in basal conditions or under adrenergic stimulation. Our data suggest a dominant effect of the D3291V variant on the RyR2 response to adrenergic stimulation.

### 3.6. Loss of Luminal Ca^2+^ Sensitivity in D3291V-RyR2 Channels

In order to characterize the variant single channel properties, microsomes isolated from HEK293 cells expressing WT and D3291V-RyR2 were incorporated into planar lipid bilayers and their activity was recorded using Ca^2+^ as the charge carrier (Figure S1a). Under this condition, the current measurement at different transmembrane potentials was indistinguishable for the WT and D3291V-RyR2 channels (Figure S1b). We found that the slope conductance (WT: 104 ± 10 pS vs. D3291V: 102 ± 12 pS) and the extrapolated reversal potentials (−30 mV) were not significantly different suggesting that the D3291V variant does not alter RyR2 Ca^2+^ conduction properties (Figure S1b). Furthermore, the identity of the HEK293-expressed RyR2 channels was confirmed by their sensitivity to the selective antagonist ryanodol, which as expected, modified the conductance and gating behavior (Figure S1c,d).

**Figure 7:**
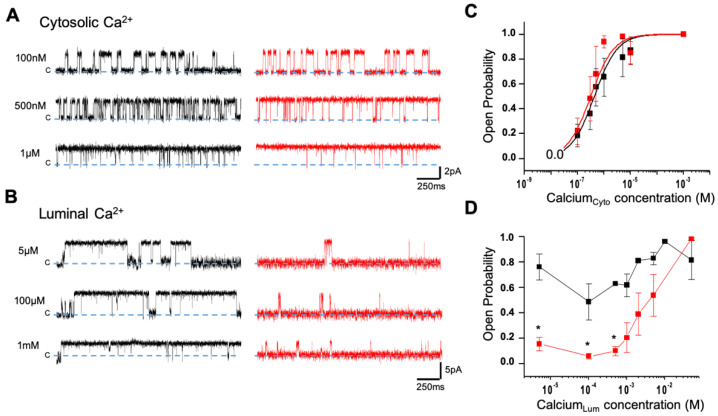
Cytosolic and luminal Ca^2+^ regulation of the D3291V-RyR2 channels: (**A**) Single-channel recordings from WT-RyR2 (black) and D3291V-RyR2 (red) were recorded at a membrane holding potential of 0 mV at three different cytosolic free Ca^2+^ concentrations. Open events are upward deflections from zero current level (blue dotted line, c). The cytosolic solution contained 120 mM Tris and 250 mM HEPES (pH 7.4), and the cytosolic Ca^2+^ levels were adjusted with 0.2 mM BAPTA and 1 mM Dibromo-BAPTA in *Cis*. *Trans* solution contained 50 mM Ca^2+^ and 250 mM HEPES (pH 7.4). (**B**) Single-channel traces from WT-RyR2 (black) and D3291V-RyR2 (red) were recorded in symmetrical conditions of 250 mM CsCH_3_O_3_S, 20 mM HEPES, pH 7.4. In *Trans*, the desired luminal Ca^2+^ levels were adjusted with 1 mM BAPTA with the cytosolic free Ca^2+^ concentration set to 5 µM in *Cis*. Channel activity was taken at +20 mV holding potential. (**C**) Open probabilities of WT-RyR2 (black squares) and D3291V-RyR2 (red squares) obtained, under the same conditions as in A, and challenged with increasing free [Ca^2+^] in *Cis*. The sigmoidal curves resulted from the fitting using a Hill function: y = Vmax × x^n^/(k^n^ + x^n^) where *Vmax* is the max velocity, *k* is Michaelis constant, and *n* is the number of cooperative sites. At the same concentrations of free Ca^2+^, the open probability of WT-RyR2 and D3291V-RyR2 channels were not significantly different. (ANOVA, one-way analysis at *p* < 0.05). The EC_50′_s for the WT-RyR2 and D3291V-RyR2 were 438 ± 75nM (n = 2–7) and 320 ± 41nM (n = 3–5), respectively. (**D**) Open probabilities of WT-RyR2 (black squares) and D3291V-RyR2 (red squares) obtained under the same conditions as in C, and challenged with increasing free [Ca^2+^] in *Trans*. The open probabilities of WT and D3291V-RyR2 channels were significantly different at low luminal concentrations of free Ca^2+^ (ANOVA one-way test at *p* < 0.05). Currents traces were filtered at 800 Hz.

To determine if the variant alters RyR2 Ca^2+^ regulation, we examined the WT and D3291V-RyR2 sensitivities to cytosolic and luminal Ca^2+^. Figure 7a illustrates the corresponding channel activity and their dependency on free Ca^2+^ concentrations. Channel activity increased when free cytosolic Ca^2+^ was increased from 100 nM to 1 µM. Figure 7c summarizes the effect of varying cytosolic Ca^2+^ on the opening probability (Po) of the channel. WT and D3291V-RyR2 channels were both maximally active (Po = 0.9) at 20–30 µM and had similar cytosolic Ca^2+^ EC_50′_s (WT: 438 nM ± 75 vs. D3291V: 320 nM ± 41). This cytosolic Ca^2+^ sensitivity data was collected at a high luminal Ca^2+^ level. However, there were differences between the two channels and their luminal Ca^2+^ sensitivity. Figure 7b illustrates the effects of different luminal free Ca^2+^ concentrations on channel activity. Contrary to their counterparts, D3291V-RyR2 channels showed decreased channel activity at 5 µM, 100 µM and 1 mM luminal Ca^2+^. This activity was characterized by low Po and short open time durations at sub-millimolar levels of luminal Ca^2+^. Inspection of the WT-RyR2 single-channel recordings showed that the duration of the open events increased to several hundreds of milliseconds as luminal Ca^2+^ was increased. In agreement with previous reports, WT and D3291V-RyR2 channels showed a reduction in single-channel conductance by millimolar levels of luminal Ca^2+^ concentration, as reflected by the amplitude of the traces (Figure 7b). Figure 7d summarizes the difference in Po between WT and D3291V-RyR2 as a function of luminal Ca^2+^ concentrations with a fixed cytosolic Ca^2+^ concentration of 5 µM. At low luminal Ca^2+^ concentration, the Po of D3291V-RyR2 channels was significantly reduced compared to WT-RyR2 channels. Taken together, these results indicated that the loss of a negative charge at the position 3291 site alters the luminal Ca^2+^ regulation of the RyR2 channel without affecting the conductance or cytosolic Ca^2+^ sensitivity.

## 4. Discussion

In this study, we identified a novel *RYR2* D3291V variant in three different families that confers an atypical CPVT phenotype. This D3291V variant is located in helical domain 2 which is outside of the classical CPVT mutation domains. To our knowledge, this is the first report of a RyR2 variant identified in both heterozygous and homozygous states. Unlike most CPVT mutations, which result in hyperexcitability of the RyR2 channels, this variant induces a loss of function phenotype, as indicated by the loss of luminal Ca^2+^ sensitivity and a blunted response to adrenergic stimulation.

### 4.1. Clinical Characterization of the Families

CPVT is a rare arrhythmia which can lead to sudden death caused by emotional or physical stress in patients without any structural cardiac abnormalities. Up to 30% of the patients experience SCD as an initial presentation, and up to 50% of them experienced cardiac arrest by age 20 to 30 in the absence of treatment [1,43,44]. Here, we reported on three families, with a CPVT diagnosis initially made only for the first one. In Family 1, the heterozygous proband had an aborted sudden death at the age of 14 while her three homozygous aunts presented with few or no syncopes. Many young patients died suddenly in the context of exercise or acute emotion; three of the second generation of Family 1 who died were possibly homozygous carriers while all the others were obligate heterozygous carriers. In Family 2, where sudden deaths occurred as well, the stress test of the proband (II.2) revealed many PVBs and ventricular bigeminy but no typical bidirectional PVT. The same variant was identified in a girl from another family who died suddenly at the age of 10 in a swimming pool. The presence of bidirectional or PVT represents an obvious indication for CPVT diagnosis, but it remains a rare observation [45]. Several studies reported that some CPVT patients who presented with SCD had a normal Holter and a negative stress test [4,45]. In our studied families, the presence of 57% of D3291V heterozygous carriers with a negative stress test, especially among the youngest, and the presence of three homozygous carriers suggests a relatively mild effect of the variant in normal daily life but contrasts with the high number of sudden cardiac deaths which occurred under adrenergic stimulation. The severity of the disease in homozygous vs. heterozygous carriers could not be determined, as we do not know if the subjects of the second generation who had cardiac arrest were homozygous or heterozygous carriers.

### 4.2. Identification of a Novel RYR2 Variant

The D3291V variant was identified for the first time at the homozygous state, but some compound heterozygous carriers have already been described [11,34,46]. This variant is located outside the four domains where most of the CPVT mutations have been identified [28]. Some CPVT variants of unknown significance (p.G2866del, p.R3190Q, p.G3037D) located in the proximity of our variant have been reported [47,48]. The variant p.Asn3308Ser, which is close to our variant, was the only one for which a functional analysis was performed, revealing a normal sensitivity to cytosolic Ca^2+^ by single channel recording, but its luminal sensitivity or its response to cAMP has not been tested [41]. This variant is more frequent in the Finns (MAF = 0.26%) than in other populations and was reported as benign.

The D3291V variant involves a highly conserved negatively charged residue among the hRyR homologs and RyR proteins from other animal species, thus reinforcing the hypothesis that this variant is associated with functional change in the hRyR2 gene product. This negative residue is conserved even in spotted gar (*Lepisosteus oculatus*), whose lineage diverged from teleosts before the teleost genome duplication (Braasch et al., 2016).

In addition, a substitution affecting the corresponding asparagine in RyR1, p.D3330G, has been described in a patient with central core disease (CDD), a neuromuscular disorder [49]. This patient was a compound heterozygous carrier of two RyR1 mutations, D3330G and G4897D [49].

### 4.3. Functional Study of the D3291V-RyR2 Channels

We performed Ca^2+^ imaging to assess the D3291V-RyR2 pathogenicity. We recorded the effect of this variant on SOICR by monitoring spontaneous RyR2 activity in HEK293 and HL-1 cells. Unlike most of the CPVT mutations expressed in HEK293 cells, we did not identify any difference between the WT-RyR2 and D3291V-RyR2 transfected cells under basal conditions [50,51]. As the symptomatic expression of CPVT is triggered by adrenergic stimulation, we challenged the transfected cells with cAMP or forskolin. The D3291V-RyR2 cells showed a weak response when treated with cAMP compared to the WT-RyR2 cells. This suggests that the D3291V variant prevents the normal response of the RyR2 channel to an adrenergic stimulation. Interestingly, Wangüemert and coworkers also reported a RyR2 variant, p.G357S, with a normal activity in basal conditions in transfected HEK293 cells but an enhanced channel activity under forskolin stress conditions [52]. In order to confirm the effect of D3291V on channel function in a more relevant physiological model, the same experiments were performed in HL-1 cells. In contrast to HEK293 cells, HL-1 is an atrial cell line with an endogenous expression of the RyR2 channel and all proteins involved in cAMP pathway [42]. As in HEK293 cells, HL-1 analyses revealed a markedly reduced response to cAMP for D3291V-RyR2 transfected cells compared to WT ones. This blunted response to cAMP in HL-1 cells allows us to suggest a dominant effect of the D3291V channels, in accordance with the numerous heterozygous carriers who experienced sudden cardiac death.

The cAMP is a well-known second messenger that activates the protein kinase A (PKA) in response to adrenergic stimulation and RyR2 channels are known to be a PKA target. Three phosphorylation sites (S2030, S2808, and S2814) have been implicated in the regulation of the RyR2 adrenergic response, even if the RyR2 phosphorylation remains a subject of controversy [53]. Our Western blot analysis of total proteins extracted from WT and D3291V-RyR2 transfected cells showed about a 40% decrease in the S2808 phosphorylation level in D3291V-RyR2 expressing cells as compared to WT cells.

Recently, it was shown that the phosphorylation domain of RyR2 embraces the catalytic subunit of PKA and results in the necessary conformational changes for PKA binding and subsequent catalytic activity. It was also shown that the CPVT-associated mutations near the phosphorylation domain can induce structural changes that may affect PKA activity [54]. Moreover, the D3291 residue belongs to the HD2 domain localized just after the phosphorylation domain 2 near the location of S2808. Therefore, we can assume that the substitution of the negatively charged Asp residue by a neutral one can cause a change in the secondary structure and affect the phosphorylation domain and therefore the activity of the PKA. Therefore, the D3291V-RyR2 may be unresponsive due to a change in conformation. Structural analysis has indicated the importance of intra/inter-domain interactions for long-range allosteric regulation of channel gating [55].

In addition, the strong in vitro effect of the mutant suggests that residues in HD2 may interact directly with the phosphorylation domain. This domain could not be crystallized in RyR2, unlike RyR1, due to intrinsic flexibility [38,56,57]. Nevertheless, RyR2 helical domains have been reported to be implicated in the conformational changes of the central domain, which are responsible for the outward tilting of S6 segments resulting in pore dilation [38].

At the single channel level, the D3291V variant did not alter the channel Ca^2+^ conductance nor its activation by cytosolic Ca^2+^, in accordance with the unchanged Ca^2+^ release in HEK293 transfected cells. In contrast, we found a reduced D3291V-RyR2 response to luminal Ca^2+^ activation. RyR2 luminal Ca^2+^ regulation mechanisms are still a matter of debate. One proposed mechanism involves Ca^2+^ binding directly to the luminal activation site [58] whereas another involves luminal Ca^2+^ passing through opened RyR2 and acting at cytosolic Ca^2+^ binding sites of the same channel [59,60,61]. A third proposed mechanism relies on Ca^2+^ binding to luminal proteins such as calsequestrin 2, which are associated with RyR2, but this complex is absent in HEK293 cells and consequently absent in our single channel experiments [62].

Since the residue 3291 is located in the cytoplasmic region of the channel and the cytosolic sensitivity is normal, the observed reduced intra-SR Ca^2+^ sensitivity would be more likely explained by an alteration of the tertiary structure modifying the exposure of a putative luminal Ca^2+^ binding site.

In summary, we identified a new CPVT RyR2 variant with a reduced Ca^2+^ luminal sensitivity and a remarkable blunted response to adrenergic stimulation. It could be classified as a LOF variant but its mechanism probably differs from the LOF RyR2 mutations recently linked to ‘RyR2 Ca^2+^ release deficiency syndrome’ [31]. Indeed, a number of our patients had positive EST and the sudden deaths all occurred in an adrenergic context in these families. When the pathogenicity of this variant has been confirmed, molecular diagnosis was undertaken for family members and mutation carriers treated with nadolol. No new cardiac event occurred under treatment.

Interestingly, reported LOF RyR2 mutations resulted in a loss or a reduction in luminal Ca^2+^ activation as occurs in our variant [32,33]. Furthermore, two murine models with heterozygous LOF RyR2 mutations inducing a loss in luminal Ca^2+^ activation, A4860G+/− and D4646A +/−, have been studied [31,63].

Zhao et coworkers, after studying A4860G+/− mice, hypothesized that the hypoactive channels would leave, at each Ca^2+^ release event through *calcium-induced calcium release* (CICR), a small quantity of Ca^2+^ in the SR. As the CICR occurred, this residual quantity of Ca^2+^ would increase. Once the threshold activation of these hypoactive RyR2 channels was reached, these channels would release Ca^2+^ and induce EADs, as was also reported for D4646A+/− murine model [31,63]. A similar mechanism could occur with the D3291V channels in cardiac myocytes. In presence of an adrenergic stimulation, the increased Ca^2+^ current I_Ca,L_, and the phospholamban phosphorylation would lead to an increased SR Ca^2+^ store. We can hypothesize that the hypoactive D3291V-RyR2 channels will not be able to regulate the Ca^2+^ store level due to their loss of luminal sensitivity.

During cAMP treatment, the amplitude of Ca^2+^ released in D3291V-RyR2 HL-1 cells decreased compared to WT cells. Furthermore, 6% of D3291V-RyR2 HL-1 cells displayed EADs only with cAMP treatment. These data are congruent with the hypothesis that Ca^2+^ may accumulate in the SR under adrenergic stress and reach the threshold of D3291V-RyR2 activation (with the consequent additional Ca^2+^ efflux) that would eventually lead to membrane depolarizations triggering potential arrhythmias.

One may question how a mutation that impairs the adrenergic response may induce arrhythmogenic Ca^2+^ waves and triggered activity. When β-adrenergic receptors are activated, the increase in cAMP activates PKA, which phosphorylates several targets that then produce enhanced Ca^2+^ entry through the LTCC and accelerate Ca^2+^ pumping to the SR through SERCA. The Ca^2+^ stored in the SR is then released as Ca^2+^ sparks or invisible Ca^2+^ leak, which are large enough to induce changes in the membrane voltage, but can limit SR overload, acting as a “safety valve”. The mutation we report lacks this “safety” mechanism. Thus, the result may be SR overload in conditions of stress. As the RyR mutant has abnormal sensitivity to luminal Ca^2+^, this store overload could be the origin of arrhythmogenic Ca^2+^ waves.

Nevertheless, the mechanisms responsible for the phenotypes associated with RyR2 variants should not be overinterpreted. The complexity is due to the central role of RyR2 channel in cardiomyocytes, its regulation by a high number of partners. In addition, recent unexpected observations, such as the electrophysiological remodeling of the surface membrane currents in the D4646+/− mice, the observed septum hypertrophy [31], as well as the post-translational modifications observed in hiPSC-derived cardiomyocytes with RyR2 mutations show that additional studies should be performed to improve our understanding of the RyR2 variants [64,65].

In conclusion, due to the cosegregation of a RyR2 variant with the disease in a large family and the long-term follow up of all family members, we were able to identify the D3291V-RyR2 variant as pathogenic. First, this shows that variants outside the CPVT hotspots, considered by the diagnostic laboratories as of unknown significance, can be pathogenic and associated with a high risk of sudden death. Secondly, the apparently normal cytosolic Ca^2+^ sensitivity and the normal Ca^2+^ conductance of the mutated channel could explain its limited severity in basal conditions and in absence of stress, thus explaining the presence of adult homozygous carriers and of numerous young heterozygous carriers with a negative stress-test. While the mutation does cause dysfunction in SR Ca^2+^ regulation, it may not be sufficient to induce a clinical phenotype in the absence of adrenergic stress. This baseline dysfunction could then be exacerbated in the context of adrenergic stress which would eventually induce typical CPVT positive stress-test with aging, or in association with modifier factors. Third, the blunted response of the D3291V-RyR2 channels to adrenergic stimuli is a new mechanism leading to sudden death. Lastly, this study points out the role of RyR2 helical domain 2 in adrenergic response and sudden death.

## 5. Limitations

We are reporting in this study a loss-of-function RyR2 mutation which is characterized by a reduced luminal Ca^2+^ sensitivity and an impaired adrenergic response in HEK293 and HL-1 cells. The absence of many cardiac-specific proteins in HEK293 cells lead us to study the effect of the variant in HL-1 cells which express RyR2 and regulatory proteins. Nevertheless, the two different cellular models are lacking the genomic background of the patient, and even though HL-1 cells express cardiac proteins, they do not completely reflect the structure and function of human cardiomyocytes. It would be interesting to generate hiPSC-derived cardiomyocytes to study the genetic background of the patient, the balance between heterozygous and homozygous carrier of this mutation, and the function of the D3291V-RyR2 in presence of its physiological partners. A murine model would be helpful to understand how such a hypoactive channel could lead to the occurrence of the typical clinical features of CPVT and the mechanisms involved in the development of ventricular fibrillation.

## Supplementary Materials

The following are available online at https://www.mdpi.com/article/10.3390/jpm11060579/s1, Figure S1: The RyR2-D3291V variant does not alter the ion channel conductance, Table S1: Clinical features of homozygous carriers and symptomatic heterozygous carriers.
