# Supplementary material for: A Type 2 Ryanodine Receptor Variant in the Helical Domain 2 Associated with an Impairment of the Adrenergic Response
Source: J Pers Med. 2021 Jun 20;11(6):579. doi: 10.3390/jpm11060579 (PMC8235491; doi:10.3390/jpm11060579)
Supplement: Supplementary file 1 [file jpm-11-00579-s001.zip › jpm-1230480-supplementary.pdf]

Supplemental files

Table S1. Clinical features of homozygous carriers and symptomatic heterozygous carriers.

Family	Subject	Gender	Genetic status	Cardiac events, age, circumstances, treatment
1	II.1	F	homozygous carrier	Several syncopes at age 20 during exercise, treated by β-blockers at age 28, and pacemaker at age 51
1	II.2	F	homozygous carrier	First syncope at age 18 during exercise, treated by beta-blockers, AF treated by electric shock at age 63
1	II.3	M	obligate homozygous or heterozygous carrier	SD at age 24 during exercise
1	II.4	M	heterozygous carrier	SD at age 54 at night, severe ischemic cardiomyopathy
1	II.5	F	homozygous carrier	no syncope, numerous PVB and PVT on Holter recording and exercise-stress test under treatment by nadolol, AF at age 62.
1	II.6	F	obligate homozygous or heterozygous carrier	SD at age 17 during exercice
1	II.8	F	obligate homozygous or heterozygous carrier	SD at age 16 in a context of acute emotion
1	II. 9	M	heterozygous carrier	AF at age 26, bigeminy at age 42, 2 syncopes at age 50 during exercise, then ICD at age 52
1	II.11	M	heterozygous obligate carrier	SD at age 48
1	III.3	M	heterozygous carrier	2 syncopes in infancy, 1 at age 22 while biking
1	III.7	F	Probably heterozygous carrier	SD at age 24 postpartum
1	III.8	M	Probably heterozygous carrier	SD at age 28 at swimming pool
1	III.9	F	heterozygous carrier, proband	Syncope at age 10 during exercise, aborted sudden death at age 14
1	III.11	M	obligate heterozygous carrier	SD at age 22 while swimming
1	III.14	M	heterozygous carrier	Syncope at age 15 at theater
1	III.16	M	obligate heterozygous carrier	SD at age 20 while swimming
1	III.17	M	obligate heterozygous carrier	SD at age 15 while biking
1	IV.13	M	heterozygous carrier	AVB at 6 months, bidirectional doublets on Holter recording, bradycardia, treated by nadolol and pacemaker
2	II.2	F	heterozygous carrier	SD at age 45, emotion

2	III.2	F	heterozygous carrier	Syncopes at the age of 20 and 25
2	III.1	M	heterozygous carrier	SD at age 7 while swimming
3	I.1	F	heterozygous carrier	SD at age 10 while swimming

SD: sudden death, AF: atrial fibrillation, PVB: premature ventricular beats, PVT: polymorphic ventricular tachycardia, ICD: implantable cardioverter defibrillator, AVB: atrio-ventricular block.

Figure S1. The RyR2-D3291V variant does not alter the ion channel conductance.

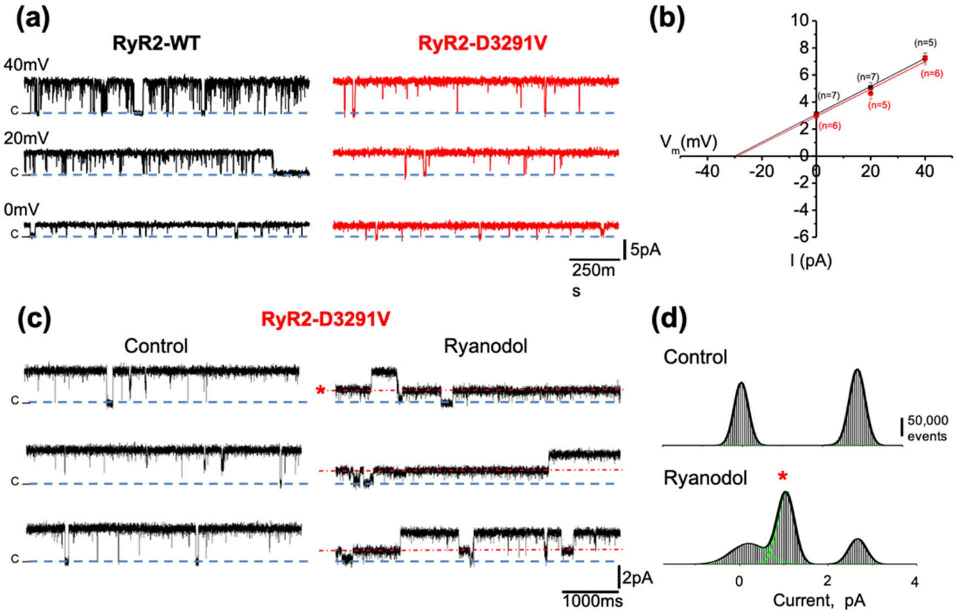

- **(a)** Single-channel recordings from WT-RyR2 (top left) and D3291V-RyR2 (top right) at different membrane holding potentials. Open events are shown as upward deflections from marked zero current level (closed state) indicated by the blue dashed line. Data were recorded in presence of 120 mM Tris/250 mM HEPES (pH 7.4) and 10 µM free Ca^{2+} in *Cis*, and 50 mM $CaOH_2$/250 mM HEPES (pH 7.4) in *Trans*. Ca^{2+} (the charge carrier) was moving in the lumen-to-cytosol direction. **(b)** Current-voltage relationships for WT-RyR2 (black squares) and D3291V-RyR2 (red circles) channels, obtained from single-channel experiments under the same conditions as in A. Slope conductances were 104$\pm$10pS and 102$\pm$12pS for the WT-RyR2 and D3291V-RyR2, respectively. **(c)** Single-channel traces from D3291V-RyR2 were obtained at 0 mV, with the same solutions used in (a). Control conditions (bottom left) show complete transitions to the open state, but in the presence of 25 µM ryanodol (bottom right) the transition to a subconductance state was favored (red asterisk). **(d)** Corresponding all-points amplitude histograms from D3291V-RyR2 single-channel recorded in C. The two

populations of events indicate the closed and open states of the D3291V-RyR2 at 10 µM free [Ca^{2+}] on *Cis*. Ryanodol addition (bottom histogram) created a new population of events that represents the subconductance state marked by a red asterisk. Current traces were filtered at 800 Hz.
